# Accounting for the Impact of Conservation on Human Well-Being

**DOI:** 10.1111/cobi.12277

**Published:** 2014-03-18

**Authors:** EJ Milner-Gulland, JA Mcgregor, M Agarwala, G Atkinson, P Bevan, T Clements, T Daw, K Homewood, N Kumpel, J Lewis, S Mourato, B Palmer Fry, M Redshaw, JM Rowcliffe, S Suon, G Wallace, H Washington, D Wilkie

**Affiliations:** * Imperial College London, Department of Life Sciences, Silwood Park CampusBuckhurst Road, Ascot, SL5 7PY, United Kingdom; † Institute of Development Studies, University of SussexFalmer, United Kingdom; ‡ Department of Geography and Environment and Grantham Research Institute on Climate Change and the Environment, London School of Economics and Political ScienceLondon, United Kingdom; § Dôl Alice, LlanthonyAbergavenny, NP7 7NW, United Kingdom; ** Wildlife Conservation SocietyBronx, NY, U.S.A.; †† School of International Development, University of East Anglia, United Kingdom, and Stockholm Resilience Centre, Stockholm UniversityStockholm, Sweden; ‡‡ Department of Anthropology, University College LondonLondon, United Kingdom; §§ Institute of Zoology, Zoological Society of LondonLondon, United Kingdom; *** Centre for Environmental Policy, Imperial College, LondonSL5 7PY, United Kingdom; ††† National Perinatal Epidemiology Unit, University of OxfordOxford, United Kingdom; ‡‡‡ Centre for Development Oriented Research in Agriculture and Livelihood SystemsP.O. Box: 2596, Phnom Penh, Cambodia; §§§ Centre for Social and Economic Research on the Global Environment (CSERGE), University of East AngliaUnited Kingdom

**Keywords:** development, ecosystem services, impact evaluation, intervention, poverty, Desarrollo, evaluación de impacto, intervención, pobreza, servicios ecosistémicos

## Abstract

Conservationists are increasingly engaging with the concept of human well-being to improve the design and evaluation of their interventions. Since the convening of the influential Sarkozy Commission in 2009, development researchers have been refining conceptualizations and frameworks to understand and measure human well-being and are starting to converge on a common understanding of how best to do this. In conservation, the term human well-being is in widespread use, but there is a need for guidance on operationalizing it to measure the impacts of conservation interventions on people. We present a framework for understanding human well-being, which could be particularly useful in conservation. The framework includes 3 conditions; meeting needs, pursuing goals, and experiencing a satisfactory quality of life. We outline some of the complexities involved in evaluating the well-being effects of conservation interventions, with the understanding that well-being varies between people and over time and with the priorities of the evaluator. Key challenges for research into the well-being impacts of conservation interventions include the need to build up a collection of case studies so as to draw out generalizable lessons; harness the potential of modern technology to support well-being research; and contextualize evaluations of conservation impacts on well-being spatially and temporally within the wider landscape of social change. Pathways through the smog of confusion around the term well-being exist, and existing frameworks such as the Well-being in Developing Countries approach can help conservationists negotiate the challenges of operationalizing the concept. Conservationists have the opportunity to benefit from the recent flurry of research in the development field so as to carry out more nuanced and locally relevant evaluations of the effects of their interventions on human well-being.

Consideración del Impacto de la Conservación sobre el Bienestar Humano

**Resumen:**

Los conservacionistas cada vez más se comprometen con el concepto del bienestar humano para mejorar el diseño y la evaluación de sus intervenciones. Desde la convención de la influyente Comisión Sarkozy en 2009, los investigadores del desarrollo han estado refinando las conceptualizaciones y los marcos de trabajo para entender y medir el bienestar humano y están comenzando a convergir con un entendimiento común de cuál es la mejor forma de hacer esto. En la conservación el término *bienestar humano* tiene un uso amplio, pero existe la necesidad de la orientación en su operación para medir los impactos de las intervenciones de la conservación sobre la gente. Presentamos un marco de trabajo para entender el bienestar humano que podría ser útil particularmente en la conservación. El marco de trabajo incluye tres condiciones: cumplir con las necesidades, perseguir objetivos y experimentar una calidad satisfactoria de vida. Resumimos algunas de las complejidades involucradas en la evaluación de los efectos del bienestar de las intervenciones de la conservación con el entendimiento de que el bienestar varía entre la gente, en el tiempo y con las prioridades del evaluador. Los retos clave para la investigación de los impactos del bienestar de las intervenciones de la conservación incluyen la necesidad de crear una colección de estudios de caso para trazar lecciones generalizables: hacer uso del potencial de la tecnología moderna para apoyar la investigación del bienestar; y contextualizar espacial y temporalmente las evaluaciones de los impactos de la conservación sobre el bienestar dentro del marco más amplio del cambio social. Existen caminos que atraviesan la confusión que rodea al término *bienestar*, y los marcos de trabajo existentes, como el del acercamiento de Bienestar en Países en Desarrollo, pueden ayudar a los conservacionistas a negociar los obstáculos de la operación del concepto. Los conservacionistas tienen la oportunidad de beneficiarse del frenesí reciente de investigación en el campo del desarrollo para así realizar evaluaciones más matizadas y relevantes localmente de los efectos de sus intervenciones sobre el bienestar humano.

## Introduction

Following the Millennium Ecosystem Assessment's focus on the importance of taking a holistic approach to people's relationships with nature (MEA 2005), there has been an increasing realization that people's livelihoods and ways of life are inextricably linked to the natural environment (McCay & Acheson 1987; Pollnac & Poggie 2008). The use of natural resources shapes people's identities as they seek to build their lives and achieve their notions of what it means to live well in particular ecological contexts (Steward 1955; Coulthard et al. 2011). As such, understanding what people mean by and aspire to for their well-being is crucial to the success of conservation measures that seek to change the relationship between human communities and the natural resource environments in which they live. But the widespread use of the term *well-being* conceals a wide variation in how the term is conceived, and this is a potential source of confusion and misunderstanding for scientists and conservation policy makers and practitioners. Such variation exists because all human beings conceive of well-being in different ways. For the concept of well-being to be useful in real-world scientific applications, we first need a generally accepted understanding of what well-being entails, for instance, its material, objective, psychological, social, and subjective elements. This then forms the basis of a framework or model, parts of which we can measure in order to understand and compare well-being outcomes for different people in different places over time. In this way, previously abstract notions of well-being can become practical scientific tools. It is important for conservationists to engage early and in a sophisticated way with debates over concepts and frameworks for understanding human well-being in order to clarify and address possible sources of misunderstanding. This will enable conservationists better to negotiate the myriad opportunities and challenges that consideration of human well-being brings.

Conservationists are engaging with human well-being for a range of reasons: to gain legitimacy in the eyes of donors, governments, and other stakeholders, including local people; because conservation outcomes often are improved if people's views and needs are taken into account (Adams et al. 2004); and to determine whether interventions are actually producing positive outcomes for people as well as nature. There has been a long-standing desire to move beyond narrow monetized approaches to the assessment of the benefits and costs of conservation. A focus on human well-being offers the prospect of taking account of a wider spectrum of gains and losses. Establishing the value and practicality of human well-being approaches to conservation is important for this evaluative role; a focus on human well-being rather than more unidimensional monetary indicators may have particular ethical weight when conservation efforts may affect peoples' ways of life and their cultures or where there may be impacts on people who are poor and vulnerable or on communities that are marginalized and lack power. Here, we consider key factors that should be taken into account when developing an understanding of the effects on human well-being of conservation interventions. We recognize the difficulties of developing a standardized well-being measure for general application and instead suggest a conceptual framework that can provide structure to discourses and support monitoring.

## Development of Well-Being as a Concept

There has been a tremendous upsurge in interest in human well-being among development researchers and practitioners over the last 2 decades. Although the term has been used philosophically and loosely for centuries, there has been a revival of social science attention to the concept and to its implications for public policy across a wide range of spheres. Interest in the relationship between environmental change, sustainable development, and human well-being was catalyzed by the Brundtland report (WCED 1987). The final report of the Sarkozy Commission on the measurement of economic performance and social progress, chaired by Amartya Sen, Joseph Stiglitz, and Jean-Paul Fitoussi, was a landmark publication (Stiglitz et al. 2009). Mindful that economic measures of development do not reflect the impact of that development on the planet or its people, one of the report's main recommendations for improving our ability to assess the quality of development progress was “… to shift emphasis from measuring economic production to measuring people's well-being” (Stiglitz et al. 2009:12).

The challenge in the report stimulated a profusion of diverse initiatives around the world to develop workable measures of progress in terms of human well-being (BRAINPOoL 2012). These measures are often based on different underpinning conceptions of human well-being (e.g., some are built on hedonic notions of happiness, whereas others are founded in eudaimonic conceptions of self-fulfillment [Ryan & Deci 2001]), and they are often intended for quite different purposes (e.g., for measurement and comparison at high levels of aggregation, such as the nation state or for policy analysis at a microscale). Despite this diversity, there is a growing convergence around a workable conception of and approach to measuring human well-being (OECD 2013).

This convergence hinges on 3 important interrelated points of agreement: that human well-being is a multidimensional phenomenon; that its assessment requires both objective and subjective measures of well-being; and that a methodology is required that is founded in some common agreements but nevertheless can be used to develop specific measures that relate to specific social and cultural contexts. The framework presented in the OECD's *How's Life?* report is a direct descendant of the thinking that was carried out under the Sarkozy Commission and represents a good example of a widely accepted framework which different countries around the world are adapting for their own specific use (OECD 2011).

Although the intended use of the *How's Life?* framework is for making international comparisons between societies, it has broadly similar foundations to the well-being framework developed by the U.K. Economic and Social Research Council-funded Research Group on Well-being in Developing Countries (WeD) (Gough & McGregor 2007). This framework was originally designed for use at a more microlevel than *How's Life?* and to be adaptable to a range of social, economic, and cultural contexts. Since its original application in 4 countries at different positions on the development trajectory (Bangladesh, Ethiopia, Peru, and Thailand), this conceptual framework and its associated research methodology have been further adapted and developed. It is particularly designed for use in communities that have high natural resource dependence (McGregor & Sumner 2010; Trimble & Johnson 2013).

One particular purpose of the WeD framework is to enable the assessment of the differential impacts of development processes on different groups in particular communities. Although it is a multidimensional framework that provides particular insights into processes of impoverishment, it is not a multidimensional poverty index of the kind developed by Alkire and Foster (2011). There are 2 important differences between the WeD framework and poverty measures such as Alkire and Foster's (2011). First, the WeD framework integrates assessments of subjective well-being alongside the assessment of objective material and human conditions. Second, it pays attention to those who are doing well, not just those who are doing badly. This is necessary because understanding who is winning and losing in development processes requires us to understand the relationships between the two. Because of its attention to these dynamics, this well-being framework is particularly suited to a critical examination of governance arrangements (Deneulin & McGregor 2010).

## Applying the WeD Framework

The WeD framework identifies 3 conditions necessary for individual human well-being: (1) when your needs are met; (2) when you can act meaningfully to pursue your goals; and (3) when you are able to experience a satisfactory quality of life (McGregor 2007). Point 1 can be objectively assessed, whereas point 3 is a subjective assessment. It is described as a social well-being framework because all aspects of human well-being depend to a greater or lesser extent on social relationships and because the different social positions of people within the same society causes them to have different experiences of well-being.

The exploration and understanding of human well-being using this framework requires a blend of quantitative and qualitative research methods and involves the investigation of objective circumstances (e.g., material wealth), social relationships, and people's subjective experiences and perceptions. Measurement of well-being may aim to elucidate the drivers of well-being or its constituents. Although some factors are obviously one or the other (e.g., income as a driver, emotional fulfillment as a constituent), there are plenty of gray areas (e.g., health as both a driver and constituent). In the WeD methodology, the collection of qualitative data is designed as an integral part of process of research. Qualitative data inform the content of quantitative inquiry and supplement the analysis of quantitative data, rather than being seen as an adjunct or residual component of the research exercise. The types of research tools used to collect these data include community profiling through participatory approaches; questionnaire surveys to assess respondents' resources and needs, income, and expenditures; interviews discussing the drivers and constituents of an individual's quality of life; qualitative analysis of the processes underpinning differences in well-being; and analyses of the structures and regimes within which people find themselves (WeD 2013).

Britton and Coulthard (2013) used the WeD framework to assess the social well-being of members of a Northern Irish fishing community. They started with a community profiling exercise in which they used a range of observational methods, key informants, and secondary sources. They then used interviews to assess individual respondents' material well-being (with a focus on resources: human [education, income], natural [fish species targeted], material [gears and boats owned], and social [e.g., membership of fishing organizations]), relational well-being (what people and organizations they had important relationships with and whether they were satisfied with the relationship), and subjective well-being (based on the Global Person Generated Index of quality of life). Their policy-relevant findings included that there was a deep frustration with current governance regimes; fishing was particularly important as a buffer to unemployment; and women had a key role in actively contributing to fisheries activities and maintaining fishing communities (this was not recognized within the EU's governance and support regime for the fishery).

## Differentiated Well-Being

The WeD approach pays particular attention to the differentiation that occurs within groups of people affected by a conservation intervention and this can be evident along lines of age, class, gender, wealth, or livelihoods. For example, when looking at fisheries conservation, it usually will be necessary to distinguish different impacts of an intervention on fishers who use different gears and to determine the nature of fishers’ traditional rights to parts of the fishing grounds. Similarly, although the frontline impacts of conservation measures may be apparent for those with the most power and influence in the community, there are almost always effects on others who are not so easily seen or heard. For example, a conservation initiative may have positive well-being impacts for men while making things more difficult for women, who often do not have a voice in communal decision making forums (Bandiaky 2008). The poor are not a homogeneous group, and some people may have a position in society that ensures that they will be least likely to benefit from changes. It is particularly important to assess well-being outcomes and processes for these groups (Maharjan et al. 2009; Daw et al. 2011).

If a well-being methodology such as WeD is effectively implemented, it should identify negative well-being outcomes as well as positive outcomes, and it must provide the opportunity for people to express negative views. In doing this, it should enable conservationists to explicitly take into account the potential for their interventions to cause harm (Bevan 2007). It should also enable a nuanced understanding to be developed of the ambiguities and trade-offs in the well-being outcomes of interventions, which may enhance well-being on some dimensions and reduce it on others.

## Well-Being Change over Time

A particular challenge for many scientists seeking to use a well-being framing for evaluative purposes is that well-being is shaped by people's ever-changing aspirations, adaptations, and social interactions; thus well-being has temporal fluidity (McGregor 2007). The development process itself is intended and expected to produce changes, not only in material conditions but also in social relationships and in value systems. Some conservation interventions deliberately seek to alter development paths, which can result in the radical reshaping of communities and changes in social structure and peoples’ aspirations for well-being. There is particular ethical sensitivity and technical difficulty for conservationists engaging with well-being in such contexts. In such situations, local perceptions of both the drivers and constituents of well-being may be significantly changed by the intervention, rendering comparisons of some dimensions of well-being with the preintervention state difficult. For example, community-based ecotourism may seek to empower local people economically, psychologically, socially, and politically (Scheyvens 1999).

Although it may be possible to gain some insight into changes in both material and relational dimensions, either by establishing a baseline for those dimensions before intervention or by recall, it is more complex to compare quality of life before and after interventions. This is because what matters to people in their assessment of their quality of life is likely to have been changed by the intervention itself. Some insight may be gained either by deeper qualitative inquiry; by drawing insights from other case studies; or from comparison of quality of life data with control sites. Long-term studies can then be designed to include, where appropriate, measurement of some constituents of well-being and aspirations currently not important locally but likely to become so in the future (e.g., vehicle ownership or Internet connections). To cope with this problem, CIFOR's Global Comparative Study on REDD used an extensive list of assets to minimize the problem of changing local perspectives leading to a change in the assets that are seen as locally relevant (Jagger et al. 2010).

Some of the effects of an intervention on well-being may be immediate, while others occur over a much longer period. Some of the consequences of responses to shocks, such as market collapse or natural disasters, have to be measured over generations rather than years; as such, it is insufficient to consider only immediately apparent effects. Conservationists should therefore prepare well-being assessments sensitive to these longer time scales. For example, an adaptive response to an intervention may not be intended primarily to maintain well-being in the short run, but represent an effort to secure the well-being of children and future generations. Of course, as the broader sustainability literature emphasises, the reverse may also be the case.

## Considering Who Well-Being Measurement Is For

Government, communities, conservation organizations, and individuals can all have very different ideas of what well-being is, how it should be pursued, and why it should be measured. Whose view prevails in evaluation exercises depends largely on power relationships. The approach taken to measure well-being, and the metrics used, will in part depend on the purpose for which the evaluation is intended; the audiences for which the data and reporting are destined; and what will be counted as evidence in each case (Schreckenberg et al. 2010). However, although there may be choices over frameworks, methodologies, and metrics, an unavoidable ethical consideration is that external definitions of well-being should not be imposed on particular people, in particular societies and cultures. A deep understanding of the contexts and the particular constituents of well-being within a community is an essential precursor to building a locally legitimate intervention.

Culturally driven aspects of well-being are particularly difficult to compare directly between locations and people. The need for context-specific detail has the potential to clash with the requirement of some donors to have externally valid measures that can be quantitatively compared between sites and projects. Although comparison at a broad-brush level may be possible, the details of locally appropriate elements of well-being are important for the design and implementation of conservation interventions in particular localities. Statistical comparisons between communities are complicated both by this issue of local detail and also by the choice of controls; affected populations may compare themselves to neighboring communities rather than those selected according to statistical comparison methods. For example, Clements (2012) evaluated the material impacts on local people of a protected area and payments for ecosystem services scheme in Cambodia using statistical matching, which produced control villages in similarly remote forested areas hundreds of kilometers away from the intervention villages. He also compared the intervention villages with villages at the edge of the protected area that were better connected to markets and services. He found that the conservation interventions had either not decreased or had increased the rate of improvement in material well-being compared with the statistically matched communities. However, villagers compared themselves with residents of villages in the buffer zone around the protected area, who were both substantially better off than them and whose well-being was increasing faster. Perceived relative change in well-being relative to one's neighbors may be more important in determining local views of conservation interventions than statistically correct comparisons with distant communities.

Evaluations of the well-being outcomes of conservation interventions may be most insightful when they include comparisons between external assessments and local perceptions. For example, when assessing the success of farmer field schools (FFSs) in East Africa at enhancing well-being through empowerment, Friis-Hansen and Duveskog (2012) collected complementary data to provide persuasive evidence for the well-being benefits of FFS; farmers’ own perceptions of their changing agency within society and external assessments of quantitative physical expressions of this agency. The use of modern technology has huge potential for empowering people to conceptualize and measure their own well-being. For example, the mobile phone application Mappiness enables people to record in real time their immediate subjective well-being as a function of their location and activities (MacKerron & Mourato 2013). The ExCites platform for mobile phones has enabled nonliterate indigenous groups to engage with timber companies in DRC and Cameroon. They can map the locations of sites of importance to their livelihoods and cultural identity and report illegal logging in real time (Lewis & Nkuintchua 2012). This approach has great potential to support the conceptualization, monitoring, and communication by local people of how their own well-being is affected by conservation interventions.

## Future Directions

It is unrealistic to expect to develop standardized metrics that can adequately measure the effects of conservation on drivers and constituents of well-being in a range of locations without consideration of local contexts (Agardy et al. 2003; Ostrom 2007; Mackinnon 2008). Conservationists need tools and approaches that reflect nuanced, context-contingent, largely self-defined conceptualizations of well-being. The adoption of a common universal framework, such as the OECD *How's Life* framework or the WeD framework, enables the generation of data that can vary in detail but can be compared in broad terms through the use of the 3 dimensions of well-being: material, relational, and quality of life. A key requirement for learning about how to apply such frameworks to the evaluation of conservation efforts will be to identify commonalities and general rules by building a collection of case studies that show how and where different approaches to measuring and enhancing well-being through conservation interventions have or have not worked.

Considering the differentiated experiences of well-being within a society can give valuable information about the choices people make and the decisions they face, as well as the winners and losers from conservation interventions. By building knowledge about the trade-offs between different dimensions of well-being that confront people and policy makers in particular contexts, conservation strategies can be designed to foster not just environmental sustainability, but also social and political sustainability (Coulthard et al. 2011). This information should allow conservationists to tune interventions to prevailing social circumstances and priorities (Schreckenberg et al. 2010). However, as circumstances change, so will people's aspirations, and the chosen incentives may no longer be attractive (Roe et al. 2012). Myriad factors other than conservation interventions can affect well-being. Honest presentation of the benefits and potential drawbacks of a proposed intervention (allowing free and prior informed consent; McShane et al. 2011) will limit the risk of raising false hopes within the community.

Conservation organizations may wish to ensure that their interventions have a positive impact on the overall well-being of the communities that they affect. However, the heterogeneity of well-being and its relationship to environmental and social change means that monitoring this impact in detail is complex. The differentiation and temporal fluidity of well-being mean that understanding the well-being impacts of conservation interventions can seem like catching smoke. However, there are already tools and frameworks available for understanding well-being, and experience is beginning to accumulate on how best to navigate the tricky path between complexity and tractability in understanding the consequences of our actions. By using change in well-being as one measure of conservation impact, we can better listen to local voices and empower marginalized groups of people to contribute to solutions that enable them to live sustainably alongside nature.
